# The Potyviral Protein 6K1 Reduces Plant Proteases Activity during *Turnip mosaic virus* Infection

**DOI:** 10.3390/v14061341

**Published:** 2022-06-20

**Authors:** Sayanta Bera, Gabriella D. Arena, Swayamjit Ray, Sydney Flannigan, Clare L. Casteel

**Affiliations:** 1 School of Plant Science, Plant Pathology and Plant-Microbe Biology Section, Cornell University, Ithaca, NY 14850, USA; rs2622@cornell.edu (S.B.); saf242@cornell.edu (S.R.); sbera@umd.edu (S.F.); 2 Laboratório de Biologia Molecular Aplicada, Instituto Biológico de São Paulo, São Paulo 04014-002, Brazil; gdiasarena@gmail.com

**Keywords:** *Arabidopsis thaliana*, defence, jasmonic acid, MPMI, papain-like cysteine proteases, phytohormones, plant-microbe interactions, plant-virus interaction

## Abstract

Potyviral genomes encode just 11 major proteins and multifunctionality is associated with most of these proteins at different stages of the virus infection cycle. Some potyviral proteins modulate phytohormones and protein degradation pathways and have either pro- or anti-viral/insect vector functions. Our previous work demonstrated that the potyviral protein 6K1 has an antagonistic effect on vectors when expressed transiently in host plants, suggesting plant defenses are regulated. However, to our knowledge the mechanisms of how 6K1 alters plant defenses and how 6K1 functions are regulated are still limited. Here we show that the 6K1 from *Turnip mosaic virus* (TuMV) reduces the abundance of transcripts related to jasmonic acid biosynthesis and cysteine protease inhibitors when expressed in *Nicotiana benthamiana* relative to controls. 6K1 stability increased when cysteine protease activity was inhibited chemically, showing a mechanism to the rapid turnover of 6K1 when expressed in *trans*. Using RNAseq, qRT-PCR, and enzymatic assays, we demonstrate TuMV reprograms plant protein degradation pathways on the transcriptional level and increases 6K1 stability at later stages in the infection process. Moreover, we show 6K1 decreases plant protease activity in infected plants and increases TuMV accumulation in systemic leaves compared to controls. These results suggest 6K1 has a pro-viral function in addition to the anti-insect vector function we observed previously. Although the host targets of 6K1 and the impacts of 6K1-induced changes in protease activity on insect vectors are still unknown, this study enhances our understanding of the complex interactions occurring between plants, potyviruses, and vectors.

## 1. Introduction

Viruses have evolved to perform all of the essential functions required to successfully infect a host, despite their very small genomes. At least two genetic strategies are observed that allow viruses to be more efficient with their limited coding potential [1]. One of the strategies is to code for proteins from overlapping open reading frames (ORF) present in the viral genome [2]. The overlapping ORFs strategy includes the presence of subgenomic RNA and a frameshift of the starting codon of an ORF [2]. Another strategy is the multifunctionality of the viral encoded proteins [3,4]. Some viral proteins are known to perform multiple critical functions in the virus infection cycle, such as genome replication, encapsidation, intercellular movement, long-distance movement, RNA silencing suppression, or vector transmission [4,5,6]. These above-mentioned strategies are not mutually exclusive and help viruses to circumvent the problem of small genome size.

The multifunctionality of viral proteins can be regulated spatially and temporally, and is dependent on ecological conditions and stages of the viral infection cycle. A study on the *Turnip mosaic virus* (TuMV) protein, nuclear inclusion a protease (NIaPro), demonstrated that apart from the proteolytic activity, it also has a role in plant-aphid interactions by re-localizing outside of the nucleus of a plant cell when the aphid vector is present [7]. Thus, [7] demonstrates the role of ecological interactions in modulating the location and function of potyviral proteins. Post-translational modification of viral proteins can also be used to change their physio-chemical properties, such as stability. For example, the coat protein (CP) of *Potato virus A* is degraded rapidly at early time points in the infection process, whereas at later stages the CP becomes more stable, when systemic infection and encapsidation are critical [8]. It was determined that phosphorylation of the CP was responsible for the dynamic stability of CP, which correlated with the viral infection cycle [8]. Another way to regulate viral protein stability is by interfering with the host’s degradation machinery. Indeed, proteins encoded by the genomes of *Cucumber mosaic virus* (CMV), *Cauliflower mosaic virus* (CaMV), *Barley stripe mosaic virus* (BSMV), *Tomato yellow leaf curl virus* (TYLCV), *Cotton leaf curl Multan virus* (CLCuMuV), and TuMV are known to interact with proteins in the autophagy or the ubiquitin-proteasome degradation pathway, affecting viral protein turnover [9,10,11,12,13,14].

Phytohormones play an important role in regulating plant–potyvirus and plant–insect interactions. For example, jasmonic acid (JA), salicylic acid (SA), and ethylene (ET) mediate plant defense responses to many phloem feeding insects such as aphids, the primary insect vector for potyviruses [15,16,17]. The JA signaling pathway is required for the induction of protease inhibitors, a protein that has been shown to reduce aphid performance on host plants [18,19]. Moreover, inhibiting the production of the phytohormones abscisic acid and ET was found to be a boon for potyvirus infection [20,21]. The potyviral protein NIaPro was found to alter ET levels and ET-related responses, and the potyviral protein HC-Pro was shown to alter SA levels [4,17,18]. The role of other potyviral proteins in regulating phytohormones and related defenses is not well understood.

Our previous work demonstrated the potyviral protein 6K1 reduces insect vector fecundity when expressed transiently in host plants, suggesting plant defenses are regulated [22]. Research related to 6K1 has been limited over the past two decades due to its small size, instability, and low protein expression levels [23,24,25,26,27]; however, early research suggested 6K1 may play a role in viral replication and in cell-to-cell movement [28,29]. More recently, [27] presented evidence that 6K1 is required during the early stages of *Plum pox virus* (PPV) replication. Another study demonstrated that the 6K2 protein, which is considered as a marker for viral replication complex (VRC), recruits 6K1 to the potyvirus replication complex [27]. Surprisingly, the 6K1 protein, which also contains a hydrophobic transmembrane domain like the 6K2 protein, is found in the soluble protein fraction, although 6K2 is found in membrane fraction [26,30].

Although using a mutated infectious clone simulates the natural viral infection process, mutating multifunctional viral proteins that are critical for virus survival can render the virus defective. In the case of 6K1, mutations are lethal for virus survival and thus, the ability to assay 6K1 for other possible functions is limited [24,25,27]. The goal of this study was to further our understanding of 6K1’s function using the model potyvirus TuMV. To circumvent the difficulty of no or reduced viral infection from mutating 6K1 in an infectious clone, we assayed the function of 6K1 by ectopically expressing it in uninfected plants and in the presence of a wildtype TuMV infection. Our results suggest jasmonic acid and papain-like cysteine proteases may play a role in changes in 6K1 stability and function during the infection process. Understanding the molecular mechanism behind the 6K1 protein degradation will pave the way for future studies on the critical multifunctionality associated with viral proteins and successful viral infection in plants.

## 2. Materials and Methods

### 2.1. Plants and Growth Conditions

*Nicotiana benthamiana* and *Arabidopsis thaliana* plants were grown in growth chambers under controlled conditions (25/20 °C day/night with a photoperiod of 14/10 h day/night) at a relative humidity of 50% and a light intensity of 200 mmol m^−2^ s^−1^. Plants were grown for 3 to 4 weeks and were used in experiments before flowering, unless otherwise noted.

### 2.2. Virus Inoculation

TuMV was propagated from the infectious clone pCAMBIA:TuMV-GFP as in [22] or from pCAMBIA:TuMV, kindly provided by Prof. Jean-Francois Laliberte. In all the experiments virus inoculation was mediated by the agro-infiltration of the agrobacterial culture after diluting it to an optical density of 0.03 at 600 nm, unless otherwise noted. The TuMV isolate of both infectious clones used in this study was UK1, World-B strain [31,32,33].

### 2.3. Plasmid Constructs

The 6K1:GFP constructs and its derivatives were produced using the Gibson cloning kit (New England Biolabs, Ipswich, UK) following the manufacturer’s instructions. Briefly, compatible gene-specific Gibson primers were designed to perform PCR and the PCR product and the digested pMDC32 vector were joined using Gibson assembly. To clone 6K1 and GFP into pMDC32 plasmid, p35:TuMV/GFP [22] was used as a template to amplify 6K1 and GFP separately. pMDC32:6K1, pMDC32:6K1:GFP, and pMDC32:GFP were then assembled as above using the Gibson kit. P19 from Tomato bushy stunt virus was cloned into pMDC32 through Gibson assembly [34].

### 2.4. Transient Protein Expression in Nicotiana Benthamiana

All the plasmid constructs were introduced into *Agrobacterium tumefaciens* GV3101 separately by heat shock and selected on LB plus 10 μg ml^−1^ of rifampicin and 50 μg ml^−1^ of kanamycin. One fresh colony was selected and grown overnight in liquid culture with the same antibiotic selection as before. The pellet of the culture was resuspended in 10 mM MgCl_2_ and 150 μM acetosyringone and left at room temperature for 2–3 h in a dark room. The solution containing the agrobacterial culture was then diluted to an optical density of 0.2 at 600 nm for transient expression experiments and at 0.4 for co-infiltrating with the TuMV infectious clone. Single leaves from 4-week-old *N. benthamiana* plants were then agro-infiltrated. After agro-infiltration, leaf tissue (100 mg) was collected 48 h post infiltration and, thereafter samples were taken at 72 h or 120 h, from separate plants and according to the individual experiment’s design. Expression was verified by microscopy, RT-qPCR and/or western blot analysis as described below.

### 2.5. Chemical Inhibitors Treatments

To investigate how the ectopically expressed 6K1 protein gets degraded, several assays with chemicals were performed that inhibit specific pathways of protein degradation in plants. MG132 (Sigma-Aldrich, St. Louis, MO, USA), and 3Methyladenine (3MA) (Tci America, Tokyo, Japan) were used to inhibit the proteasomal degradation and autophagy pathways, respectively, and E64 (Sigma-Aldrich, St. Louis, MO, USA) was used to inhibit the cysteine proteases. The 3MA (10 mM) solution was prepared by dissolving it in phosphate buffered saline (PBS) containing 2% DMSO. MG132 (50 μM) was prepared in PBS and E64 (50 and 100 μM) was prepared in water. Forty-eight hours after 6K1 agro-infiltration, chemical inhibitors (1 mL) were infiltrated in the previously agro-infiltrated leaves. Post agro-infiltration samples were collected at 60 hpi.

### 2.6. Western Blotting

Leaf tissue was collected and flash frozen in liquid nitrogen. Later it was crushed in a lysis buffer (10 mM sodium citrate, 1% SDS, 30 mM NaCl, 0.4% 2-mercaptoethanol, 2X EDTA-free protease inhibitor cocktail) and boiled for 10 min in a 1.5 mL tube. The supernatant was mixed with an equal volume of loading dye and fractionated by a 12% SDS–polyacrylamide gel electrophoresis gel under reducing conditions. Afterwards protein bands were transferred to a nitrocellulose membrane using a transfer apparatus according to the manufacturer’s protocols (Bio-Rad, Hercules, CA, USA). After incubation with 5% nonfat milk in TBST (50 mM Tris-Cl, 150 mM NaCl, 0.1% TWEEN 20) for 2 h, the membrane was incubated with an antibody against GFP (1:5000 dilution) for 2 h at room temperature. The GFP antibody was already conjugated to the horseradish peroxidase (Anti-GFP-HRP, http://www.miltenyibiotec.com, #130-091-833, accessed on 1 June 2017). Blots were washed with TBST three times for 15 min each and developed with an enhanced chemiluminescence system according to the manufacturer (Bio-Rad, Hercules, CA, USA). However, when 6K1 was transiently expressed we did not always detect 6K1 at 58 hpi. As the 6K1 protein is prone to degradation, we increased the protease inhibitor cocktail concentration in later extractions, which improved stability at 58 hpi.

### 2.7. Quantification of RNA

Total plant RNA extraction and DNAse treatment were performed using the SV Total RNA Isolation Kit (Promega, Madison, WI, USA), and cDNA was synthesized using Oligo dT from 1 μg of total RNA. Viral RNA and GFP transcripts were quantified relative to the actin transcripts using reverse transcription quantitative real-time PCR (RT-qPCR). All the primers used for quantification are listed in Table S1. RT-qPCR was performed using the Bio-Rad CFX384™ Real-Time System in a 10 μL mixture containing SYBR Green PCR Master Mix (Applied Biosystems, Foster City, CA, USA). The thermocycling conditions were: 2 min polymerase activation at 50 °C followed by initial denaturation for 2 min at 95 °C and 45 cycles at 95 °C for 15 s, 60/55 °C for 1 min. Each sample was quantified in triplicates and no template control was included. Cycle time values were automatically determined for all plates and genes using the Bio-Rad CFX384™ Real-Time System software. Analysis of RT-qPCR fluorescence data was performed and expressed in fold change relative to actin using the ΔΔCT method [35].

### 2.8. RNA-Seq Experiment

Wild-type Arabidopsis (*Arabidopsis thaliana*) Columbia-0 were obtained from the Arabidopsis Biological Resource Center (http://www.arabidopsis.org, accessed on 1 June 2014). After 3 weeks of growth, one-half of the plants was infected with TuMV-GFP as described above. After one week, infected plants were identified by fluorescence under UV light. For aphid induction, 15 adult apterous aphids were caged on one leaf per plant for six uninfected plants and six infected plants. A corresponding set of six infected and six uninfected plants received cages with no aphids as controls for aphid feeding. Caged leaves were developmentally matched, and infected leaves were verified for full infection before caging based on GFP visualization. Forty-eight hours after aphid placement, cages and aphids were removed and leaves were pooled for every two plants resulting in three replicates for each treatment. RNA was then extracted as described above.

### 2.9. Library Preparation, and Sequencing

Sequencing libraries were prepared using a multiplexing library protocol [36]. Briefly, oligo(dT) Dynabeads were used to purify mRNA, which was then fragmented, and the first-strand cDNA was synthesized using random primers, dNTP, and reverse transcriptase. The second-strand was synthesized using a dNTP mix, DNA Polymerase I, and RNase H, ends repaired, and adenylated. The cDNA fragments were ligated to adapters, selectively enriched by PCR, and purified using the AMPure XP beads. The library quality was assessed using the Agilent Bioanalyzer 2100 system and sequenced using an Illumina HiSeq 2000 instrument.

### 2.10. RNA-Seq Data Analysis

The quality of the raw reads was assessed with FASTQC and ShortRead. All samples presented reads with high quality. Reads were mapped against *A. thaliana* TAIR10 genome using TopHat2 [37]. The number of reads per gene were counted using HT-Seq and normalized using the normalization method implemented inside the edgeR Bioconductor package. The clusterization profile of the normalized samples was verified by Principal Component Analysis (PCA) and Spearman correlation. Differential expression test was conducted using edgeR, according to [38], using mock-infected samples as the reference control treatment. Genes with an FDR-corrected *p*-value lower than 0.1 were considered as differentially expressed genes (DEGs). Reads are available at the NCBI SRA (PRJNA60524).

### 2.11. Gene Set Enrichment Analysis (GSEA)

To identify molecular mechanisms potentially relevant to the plant response to TuMV and aphids, a GSEA was conducted. The GSEA identified biological processes (BPs), molecular functions (MFs), and cellular components (CCs) that were over-represented among a list of DEGs. Categories with a *p*-value lower than 0.005 in a hypergeometric test were considered enriched.

### 2.12. Protease Activity Assays

Twenty *N. benthamiana* plants that were 4-weeks old were agro-infiltrated with TuMV as described earlier. Twenty plants of the same age were left uninfected as a control. Five days post inoculation, the third youngest leaf of ten infected and ten uninfected plants were each agro-infiltrated with the 6K1:GFP or GFP control construct, and 100 mg of plant tissue were collected 60 h post inoculation. To evaluate the effect of proteases in virus infected plants in early and late stages of infection, twenty four 4-week old plants were also co-infiltrated with TuMV and either GFP or 6K1:GFP (twelve plants for each treatment), and tissues were collected after 60 h from local and systemic leaves. Plant tissues were homogenized in 1 ml of 0.046 M Tris-HCl and 0.0115 M CaCl_2_ buffer (pH = 8.1) with 5% polyvinylpolypyrrolidone. The homogenized samples were incubated on ice for 10 mins followed by centrifugation at 11,000× *g* for 10 mins at 4 °C. The supernatant containing the soluble proteins from the leaves were then used for assays. Total protein extracted in each sample was measured by Bradford assay (Bradford, 1975). Fifty microliters of the protein extract were used to measure total protease activity in each sample using FITC Casein according to manufacturer’s protocol (Sigma Aldrich). Known concentrations of trypsin were used as standards for protease assay. Protease activity in each sample was reported as equivalent amount of trypsin activity per mg of total protein.

### 2.13. Statistical Analysis

The distribution of all values for all variables was analyzed to test for normality using the Shapiro-Wilk test [39] and was also tested for homogeneity of variances using the Levene test. To determine if 6K1 expression impacts virus infection in local and systemic leaves (Figure 5A,B), the data were analyzed by generalized linear models (GLM) with a normal distribution curve which fitted the observed data. The model included treatments (TuMV, GFP, 6K1:GFP) and leaves (local, systemic) as fixed factor in a full factorial model. The GLM analysis was selected because it is a robust method with respect to the distribution of the data and allows contrasting both balanced and non-balanced models. To determine if the observed differences between classes of the same factor were significant, least significant difference (LSD) analysis were performed. The data related to Figures 2, 4 and 5C,D were analyzed either by *t*-test or Kruskal-Wallis test. To determine if the protease activity between 6K1:GFP and GFP treated plants were different from each other (Figures 2D and 5C,D), the data were log-transformed to meet assumptions of normality and a one-way ANOVA was performed using R. The statistical analyses were performed using the SPSS v.24.0 program (SPSS Inc., Chicago, IL, USA) or R (R Core Team, 2017).

## 3. Results

### 3.1. 6K1 Expression Inhibits Transcripts Related to Jasmonic Acid Biosynthesis and Protease Inhibitors

**Figure 1:**
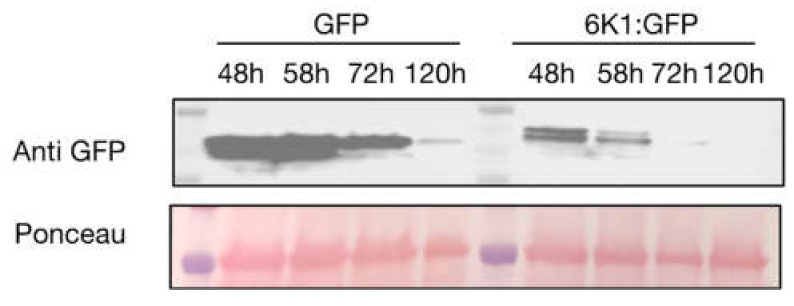
Ectopic expression of 6K1:GFP in *Nicotiana benthamiana*. The constructs GFP and 6K1:GFP were agroinfiltrated in *N. benthamiana* leaves. Western blots were performed with proteins extracted from the agroinfiltrated leaves collected over time. Anti-GFP antibodies were used in both western blots and Ponceau staining was performed to check for loading control. The top band in the 6K1:GFP blot represents 6K1 fused GFP. All western blots are representative of at least two replicates which each contained 3 plants per treatment.

As our previous results demonstrate 6K1 expression in hosts has a negative impact on aphid vectors compared to control [22], we hypothesized that 6K1 might alter jasmonic acid signaling, and related plant defenses to aphids, such as the production of protease inhibitors [40]. To address this, we first examined the stability of 6K1 using transient expression in *N. benthamiana*. Thus, the kinetics of GFP and 6K1:GFP accumulation were monitored by western blot analysis using antibodies that recognize GFP (Figure 1). For GFP, protein expression was stable until 72 hpi, whereas, for 6K1:GFP, protein expression was stable until 58 hpi (Figure 1).

**Figure 2:**
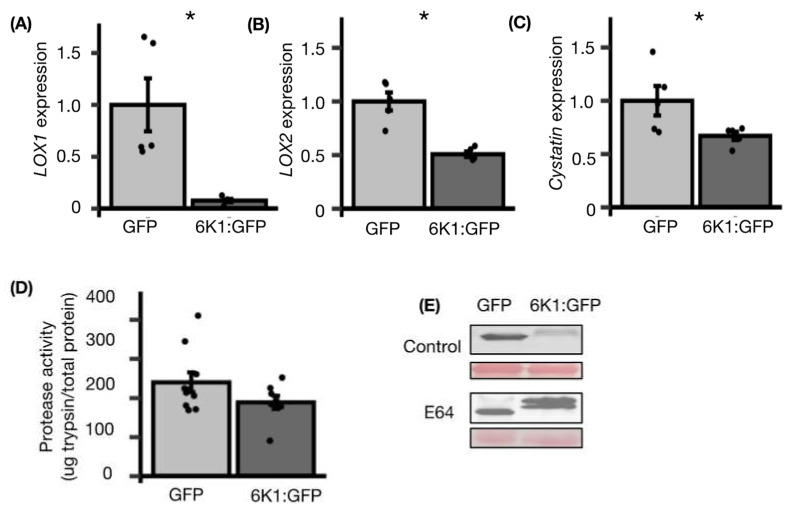
6K1 expression inhibits transcripts related to protease inhibitors and jasmonic acid accumulation. RNA was extracted from *N. benthamiana* leaves transiently expressing the GFP or 6K1:GFP. Transcript abundance was measured using qRT-PCR of: (**A**) *LOX1* and (**B**) *LOX2*, related to jasmonic acid biosynthesis; and of (**C**) *Cystatin*, a protease inhibitor. The relative quantification was performed by using actin as reference gene and GFP treatment as the calibrator. Each result is the mean from 5 replicated plants ± SE. The stars (*) denotes if the mean values were significantly different at *p* < 0.05 as determined from either *t*-test or Kruskal-Wallis test. (**D**) Samples were collected from leaves transiently expressing the GFP or 6K1:GFP, and protease activity was quantified. One-way ANOVA was used to determine there was no significant difference between means (n = 10; non-significant; mean ± SE). (**E**) The GFP or 6K1:GFP constructs were agroinfiltrated in *N. benthamiana* leaves with and without a cysteine protease inhibitor, E64. Proteins were extracted and SDS-PAGE gels were run with an equal volume of each sample (9 μL). Anti-GFP antibodies were used in both western blots and Ponceau staining was performed to check for loading control. The western blot is representative of at least two replicates which each contained 3 plants per treatment.

Next, we measured the abundance of two transcripts related to JA biosynthesis, *LIPOXYGENASE1* (*LOX1)* and *LIPOXYGENASE2 (LOX2*), in *N. benthamiana* leaves after 48 h of transiently expressing the GFP or 6K1:GFP protein. 6K1:GFP expression significantly inhibited *LOX1* and *LOX2* transcripts compared to controls (Figure 2A,B). To determine if protease inhibitors are also decreased in plants expressing 6K1, we measured transcript abundance of the *N. benthamiana Cystatin* protease inhibitor (Niben101Scf00862g02050.1). The reason we chose to focus on a cystatin was because aphids have acidic guts, and cysteine proteases have been shown to be one of the most active proteases under low pH and in aphid guts [41,42]. This *Cystatin* has the highest identity to a tomato cystatin that was previously implicated in plant-insect interactions [43]. Relative to the control treatment (GFP), *Cystatin* transcripts were significantly reduced in the presence of 6K1:GFP (Figure 2C). These results could indicate that 6K1 is inhibiting JA-dependent defenses and that other plant defense mechanisms are important for the anti- vector impacts of 6K1s that we observed previously [22].

### 3.2. The Ectopically Expressed 6K1 Protein Is Degraded by Cysteine Proteases

Protease inhibitors contribute to protein regulation in plants by preventing protein turnover during development and senescence [44]. We hypothesized protease inhibitors may also be reduced, which may contribute to the increased 6K1 protein turnover when expressed in *trans* as shown here and in previous reports [26]. To address this we first measured the total protease activity of plants transiently expressing GFP or 6K1:GFP. In this experiment, transient expression of the 6K1:GFP protein had no impact on total protease activity of the plant compared to the GFP control (Figure 2D). As the substrate used in this assay, Casein, is hydrolyzed by many proteases, we could not discount the fact that there still may be differences in cysteine proteases specifically. To address this we next used a cysteine protease chemical inhibitor, E64, in plants expressing GFP and 6K1:GFP and conducted western blots. E64 increased accumulation of the 6K1:GFP protein significantly relative to the GFP control (Figure 2E), demonstrating cysteine protease are involved in 6K1 turnover.

To further investigate the instability of the 6K1:GFP relative to the GFP protein, chemical inhibitors were next used that target the different protein degradation pathways: 3MA (autophagy inhibitor) and MG132 (proteasomal inhibitor) (Figure S1). 3MA and MG132 did not impact 6K1:GFP protein accumulation relative to the GFP control. Next, to assay if 6K1 protein accumulation is affected at the level of its mRNA stability, an experiment was performed with a viral suppressor of RNA silencing (VSR), P19, co-infiltrated with GFP/6K1:GFP. Western blot analysis and *gfp* transcript abundance revealed that 6K1:GFP accumulation was higher in the presence of P19 in comparison to the control (Figures S1 and S3).

### 3.3. Transcriptome Wide Analyses Revealed That Aphid and TuMV Differentially Affect Host Protein Degradation Pathways in A. thaliana

The results above suggest protease inhibitors and protease may have pro- and anti-viral roles during TuMV infection, respectively. Previous studies demonstrated a critical role of autophagy and proteasomal pathway in potyviral protein regulation; thus, to investigate if either of these pathways may play an important role in the degradation of 6K1, we next conducted RNAseq. We analyzed the transcriptome of *A. thaliana* plants with and without TuMV infection and aphid-vector infestation so that we could examine multiple transcripts and pathways at the same time. TuMV and its vector (e.g., aphids) have a wide host range that consists of *A. thaliana*, as well as *N. benthamiana*. Moreover, the accessibility to a high number of computational resources related to *A. thaliana* allowed us to investigate our targets as well as additional protein degradation pathways more thoroughly. Differential gene expression analysis revealed TuMV infection had a greater impact on transcriptional changes compared to aphid infestation (Figure S2A–C). Overall, 188 and 368 genes were differentially expressed exclusively in response to aphid and TuMV treatments, respectively, whilst 19 genes were regulated in both treatments (Figure S2B, FDR-corrected *p*-value lower than 0.1). Only 15 transcripts were shared among all treatments, and the greatest number of transcripts were regulated in the treatment with both aphids and TuMV compared to controls (Figure S2C).

Gene set enrichment analysis (GSEA) was used next to determine which biological processes were over-represented in each treatment (Tables S2–S4). For aphid treatment, categories related to JA and abscisic acid, along with related processes were enriched (Table S1), which are in line to our previous published data [16,40,41]. For TuMV treatment, GSEA analysis indicated biological process related to salicylic acid biosynthesis and responses to JA and ethylene were significantly enriched (Table S2), which were also consistent with our previous findings [21,45]. Although we observed that TuMV infection caused the most substantial changes in gene expression (Figure S2), it was connected to fewer biological processes (130), compared to aphids (188). Nevertheless, the highest number of biological processes were found to be regulated when both TuMV and aphids were present (230, Table S3).

**Figure 3:**
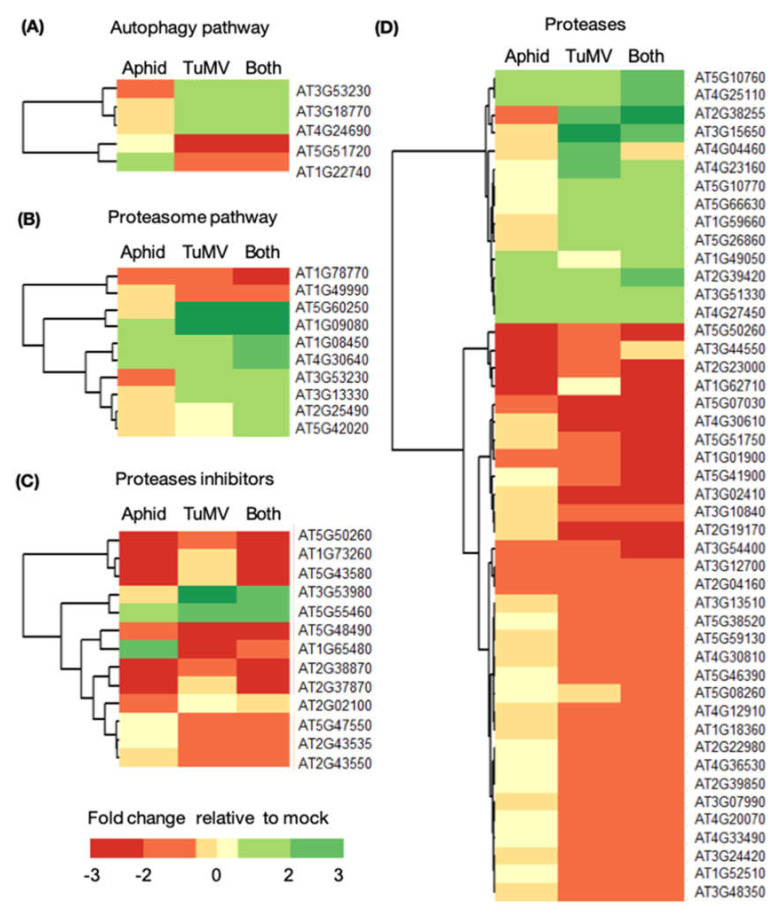
TuMV infection and aphid feeding significantly impacts protein degradation pathways in *A. thaliana*. RNA-seq was performed with mock-inoculated *A. thaliana*, *A. thaliana* one week after TuMV infection (TuMV), *A. thaliana* 48 h after infestation with the *Myzus persicae* aphid (Aphid), a vector of TuMV, or from plants with both treatments (Both). Each sample represented a pool of two plants and three samples were taken per treatment (N = 3, 6 plants total). Heatmaps show genes that where at least 1.5 times differentially expressed relative to mock (*p*-value < 0.1). Differentially expressed genes were grouped according to the following protein degradation pathways: (**A**) Autophagy, (**B**) Proteasome, (**C**) Protease inhibitors, and (**D**) Proteases.

To study the impact of treatments on protease inhibitors/proteases and specific protein degradation pathways (autophagy and proteasome), we next searched the transcriptome for transcripts whose levels changed > 1.5 times (upregulated or downregulated; *p* < 0.1) relative to the mock in either of the treatments (Figure 3). Our data shows TuMV, aphids, or both treatments regulated five genes related to autophagy, 10 genes related to the proteasome, 14 related to protease inhibitors, and 46 related to proteases (Figure 3A–D). Specifically, TuMV significantly induced the expression of *NBR1* (AT4G24690), which was shown to be up-regulated previously by TuMV and to have a pro-viral function [9]. The autophagy and proteasome pathways were found to be most differentially regulated when either TuMV or both TuMV and aphids were present, and least when aphids were present alone. Aphids alone and aphids with TuMV had the greatest impact on protease inhibitor genes among all the treatments (Figure 3C). The greatest number of genes related to proteases were significantly regulated when TuMV was present either alone or with aphids, whereas only aphids did not have as large of an impact on proteases (Figure 3D). Taken together these results suggest TuMV infection downregulated mostly protease genes and upregulated some autophagy and proteasome related genes, although aphid feeding was mostly associated with the downregulation of protease inhibitor genes (Figure 3A–D).

### 3.4. TuMV Infection Increases 6K1 Protein Stability and 6K1 Decreases Protease Activity

**Figure 4:**
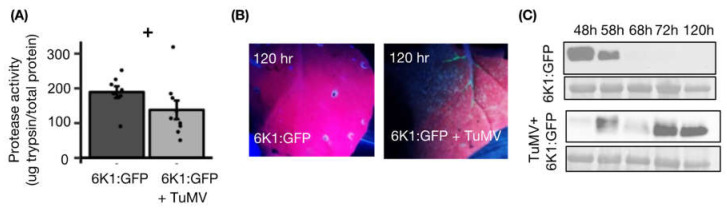
TuMV infection decreases plant protease activity and increases 6K1 stability. *N. benthamiana* leaves were agro-inoculated with TuMV and 6K1:GFP or just 6K1:GFP. (**A**) Protease activity was quantified and 6K1:GFP quantified with (**B**) UV light at 120 hpi and (**C**) western immunoblots over time. For each sample, an equal volume (10 μL) was loaded into each well of an SDS-PAGE gel. Anti-GFP antibodies were used in both western blots and Ponceau staining was performed to check for loading control. Each result is mean from ten biological replicates in (**A**). All western blots are representative of at least two replicates which each contained three plants per treatment. A one-way ANOVA was used in (**A**) to check for significance among means (*n* = 10, mean ± SE, + indicated a *p*-value of <0.1).

Our transcriptome analysis results suggest TuMV infection may have altered the protease activity of plants possibly to increase 6K1 stability. To test this, we expressed 6K1:GFP with and without an infectious clone of TuMV in *N. benthamiana* and measured total protease activity at 120 hpi. Total protease activity was reduced when 6K1:GFP was expressed in the presence of TuMV compared to without TuMV (Figure 4A). In this experiment, 6K1:GFP was not detected by UV light when expressed alone (Figure 4B), and 6K1:GFP was visible with UV light expressed in the presence of TuMV in plants 120 hpi (Figure 4B). Next, the kinetics of the 6K1:GFP protein with and without TuMV present were monitored by western blot analysis using antibodies that recognize GFP. Without TuMV infection, a band was detected at 48 and 58 hpi and had an estimated molecular weight of 33kD, which is around the expected size of the 6K1:GFP fusion (Figure 4C). At the 68 hpi time point and later 6K1:GFP expressed alone was not detected in the western blot analysis (Figure 4C). In contrast, 6K1:GFP in the presence of TuMV was detected at all time points in western blot analysis (Figure 4C). At early time points (48 and 58 hpi), protein levels of 6K1:GFP were reduced in the presence of TuMV compared to without the infectious clone (Figure 4C). As a control we conducted a similar experiment using GFP with and without TuMV (Figure S4A) and found similar results. To investigate the role of protease activity in increased GFP stability, we next measured the total protease activity in infected and uninfected plants expressing free GFP (120 hpi; Figure S4B). Total protease activity was not significantly different when GFP was expressed in the presence of TuMV compared to without TuMV at 120 hpi (Figure S4B). As our previous findings suggest that *6K1:gfp* transcripts are not stable upon transient expression (Figure S3), we next measured *gfp* transcript abundance of TuMV-infected leaves transiently expressing GFP or 6K1:GFP. In the presence of TuMV, the *6K1:gfp* transcripts accumulation was still significantly less relative to the *gfp* transcripts (Figure S4C). Overall, these results suggest that TuMV increases 6K1 and GFP stability over time, that increased 6K1 stability is not mediated by increasing the stability of *6K1:gfp* transcripts, and that high 6K1 stability results in reduced plant protease activity in the presence of TuMV.

### 3.5. 6K1:GFP Expression Inhibits Plant Protease Activity in Infected Leaves and Increases TuMV Accumulation in Systemic Leaves

**Figure 5:**
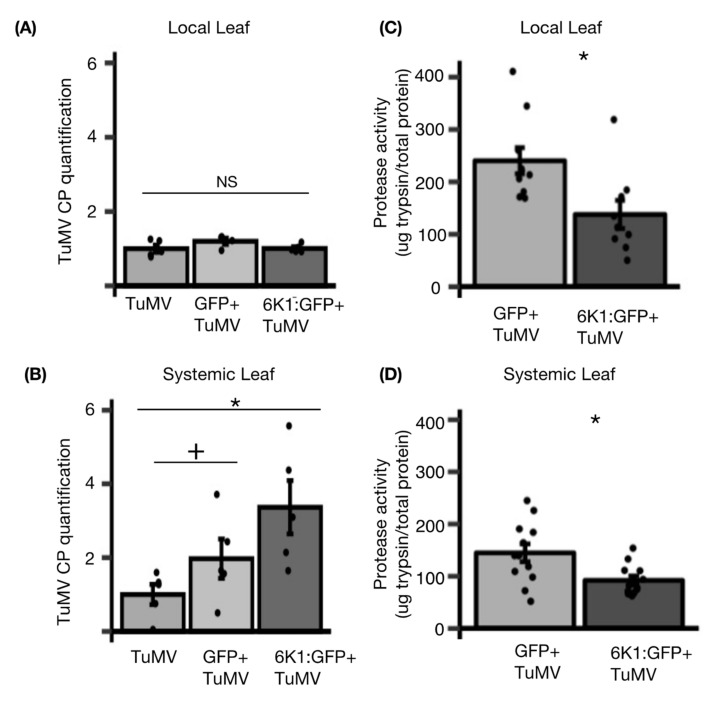
6K1:GFP expression inhibits plant protease activity in infected plants and increases TuMV accumulation in systemic leaves. (**A**,**B**) GFP or, 6K1:GFP constructs were co-infiltrated with TuMV into *N. benthamiana* leaves. In the control treatment, only TuMV was agro-infiltrated. At 60 h post infiltrations, CP gene-specific primers were used for the quantification of viral RNA relative to the actin in the local (**A**) and systemic leaves (**B**). (**C**,**D**) In a separate experiment GFP and 6K1:GFP were agroinoculated into local leaves with TuMV (**C**) or in systemically infected leaves (**D**) and protease activity measured. Significance was determined using differences at a *p*-value of * < 0.05 and + < 0.1 as determined from a least significance difference (LSD) test (*n* = 5, mean ± SE; GLM performed for **A** and **B**; *n* = 10, mean ± SE; one-way ANOVA for **C** and **D**). NS indicates non-significant (1 A).

To determine the impact of transiently expressed 6K1 on virus accumulation, leaves were infiltrated with either TuMV, GFP + TuMV, or 6K1:GFP + TuMV, and then the abundance of TuMV CP transcripts was quantified in local and systemic leaves. Virus CP transcripts accumulated to a similar level in local leaves in which only TuMV was infiltrated compared to leaves infiltrated with GFP + TuMV or 6K1:GFP + TuMV (Figure 5A). In systemic leaves, greater amounts of TuMV CP transcripts were detected when 6K1:GFP was co-infiltrated with TuMV (6K1:GFP + TuMV) relative to the TuMV or GFP + TuMV treatment (Figure 5B). To further investigate the role of protease activity in 6K1 pro-viral role, we next measured the total protease activity in local and systemically infected *N. benthamiana* leaves transiently expressing GFP or 6K1:GFP (Figure 5C,D). In both systemically and locally infected leaves, transient expression of the 6K1 protein significantly inhibited plant protease activity compared to the GFP controls (Figure 5C,D). These results suggest while 6K1 expression increases, a higher amount of TuMV accumulates in systemic leaves, possibly due to the impacts of decreased plant protease activity on 6K1 stability.

## 4. Discussion

In this study, we demonstrate that the stability of the 6K1 protein is dynamic and increases over time in the presence of TuMV (Figure 4). Decreased protein stability of the ectopically expressed 6K1 was due to cysteine proteases and inhibition of cysteine protease inhibitors (Figure 2 and Figure S1). It was previously reported that the expression of 6K1 protein *in-vivo* is quite low and affinity purification was required to detect PPV’s 6K1 protein during viral infection [25]. In our study transient expression of the 6K1 protein was lowest at 48 hpi, relative to other timepoints in virus-infected plants and increased overtime (Figure 4 and Figure S5). A similar observation was reported in [26] where the 6K1 protein was not detected at 48 hpi in virus-infected plants, but at 96 hpi they were able to detect it. It is important to note when a second GFP-tagged copy of PPV’s 6K1 was expressed in *cis* from an infectious clone, increased stability of 6K1 was not observed overtime [26]. It is tempting to speculate that early in the potyvirus infection process the 6K1 protein may be inhibited by the virus or plant, although later in the infection process 6K1 stability is increased, enabling new functions. For example, we demonstrated ectopic expression of 6K1 increased viral accumulation in systemic leaves (Figure 5), suggesting 6K1’s increased stability may play a role in regulating viral movement.

The increase in *trans* 6K1 protein accumulation in TuMV-infected plants might be due to multiple changes in plant pathways. We observed a higher accumulation of the transiently expressed 6K1 protein in the presence of the VSR P19, which suggests the 6K1 mRNA is unstable and a target of host RNA silencing machinery (Figures S1 and S4C). As the activation of host RNA silencing machinery is one of the primary defense responses that target viruses [46,47], it seems reasonable that parts of the viral RNA genome will be potent activators of RNA silencing. Our observation is in line with Nigam et al., 2020, which reported high amounts of siRNAs derived from TuMV’s 6K1 and CI RNAs sequence are detected during infection [48]. Nevertheless, potyviruses also encode for VSRs (HCPro and VPg) to inhibit the host RNA silencing pathway and ensure successful virus infection [4,49]. The VSR HCPro is multifunctional and can also regulate the salicylic acid and autophagy pathways [50]. Thus, the underlying interactions that may mediate increased 6K1 stability during TuMV infection may be similarly complex. We also observed transient protein accumulation of both GFP and 6K1:GFP was reduced at 48 h to 58 h in the presence of TuMV, compared to without (Figure 4C and Figure S4C). The potyviral genome and VPg sequester eIF4E/eIF(iso)4E for viral translation during infection, which in turn might reduce the availability of free eIF4E/eIF(iso)4E to translate other mRNA transcripts, such as the transiently expressed GFP and 6K1:GFP [51,52]. At later time points we observed the opposite pattern, transient protein accumulation of both GFP and 6K1:GFP increased at 72 h to 120 h in the presence of TuMV, compared to without (Figure 4C and Figure S4C). This might be due to an increased accumulation of HCPro and VPg, which both are known to have RNA silencing suppression activity and thus, more GFP and GFP:6K1 transcripts may be available for translation for longer time periods [11,53,54].

Papain-like cysteine proteases (PLCPs) have been shown to play an important role in host defenses against many pathogens including viruses [55,56,57]. We show here 6K1 protein accumulation increased in the E64 treatment, a chemical inhibitor of PLCPs, demonstrating cysteine proteases have a role in degradation of the ectopically expressed 6K1 protein (Figure 2 and Figure S1). Many PLCPs are localized in autolysosomes, suggesting additional studies on the role of the autophagy pathway in the degradation of the 6K1 protein will be required [58,59]. We go further and demonstrate that protease activity, and PLCPs inhibitors (cystatins) are regulated by 6K1 and during TuMV infection (Figure 2, Figure 3 and Figure 5). The inhibitory role of cystatins against potyvirus infection is quite well known and functional recombinant cystatins were engineered to produce potyvirus-resistant crops [60,61]. Thus, the suppression of *Cystatins* transcripts by 6K1 may be a counter defense and an indirect way of promoting TuMV infection (Figure 2 and Figure 5).

Previous studies have shown that 6K1 has a role in viral replication and may mediate cell-to-cell movement [26,27,28,62]. Our data provides evidence of a novel function associated with the 6K1 protein i.e., inhibition of JA biosynthesis transcripts (Figure 2). It is well established that phytohormones mediate many different components of plant-virus-insect interactions and may regulate virus transmission [40,63,64,65,66,67,68]. Inhibition of JA in the presence of 6K1 protein may indicate a possible role of 6K1 in mediating ecological interactions. Indeed, it was shown that 6K1 decreases aphid fecundity [22], and aphids induce JA in plants [21]. Aphid induction of JA may be beneficial for aphids through the suppression of SA-dependent defences responses. Consistent with this hypothesis, mutations in a fatty acid desaturase 7 (FAD7), an essential component for generating JA precursors, increases salicylic acid accumulation and reduced aphid performance [69]. Thus 6K1 may be inhibiting JA and preventing JA-inhibition of SA and related defences, which may make the plant less desirable for aphids.

As viral proteins are often associated with more than one function, it is critical to assay their function throughout the virus infection cycle and under different ecological conditions. Our results support earlier observations of 6K1′s role in systemic movement, using ectopic expression with and without the virus instead of mutating an infectious clone (Figure 5), and suggest the transiently expressed 6K1 protein retains its native function. As viral RNA acts as an open reading frame and may also form a functional RNA element, mutating a part of the viral genome can be detrimental to virus survival [70,71]. The evolution of potyviruses with plants has progressed for about 15,000 to 30,000 years and the emergence of new potyviral species are still being documented [72,73]. Further, potyviruses are among the most widely-distributed pathogens in crops, hampering the production and the quality of food [74,75,76]. Although our study paves the way for more thorough investigation of the 6K1 protein, its multifunctionality, and its role in plant-virus-aphid interactions, a thorough understanding of the underlying molecular mechanism causing potyviruses disease will be required to develop innovative intervention strategies and prevent viral epidemics in the future.

## Data Availability

Reads are available at the NCBI SRA (PRJNA60524), and all other data is available upon request.

## Supplementary Materials

The following supporting information can be downloaded at: https://www.mdpi.com/article/10.3390/v14061341/s1, Figure S1: Western blot analysis of transiently expressed GFP and 6K1:GFP in the presence of chemical inhibitors, Figure S2: Differential gene expression analysis of *A. thaliana* transcriptome; Figure S3: Post-transcriptionally 6K1:GFP transcript accumulation was affected; Figure S4: TuMV increases GFP stability, but does not affect protease activity, Figure S5: A histogram indicating the protein band intensities observed in Figure 4C; Table S1: A list of primers used for quantification. Table S2: Enriched biological processes (BPs) during the *A. thaliana* interaction with *Myzus persicae* aphids. Table S3: Enriched BPs during the *A. thaliana* interaction with turnip mosaic virus (TuMV). Table S4: Enriched BPs during the *A. thaliana* interaction with a combination of both *M. persicae* aphids and TuMV.
