# Supplementary material for: The Potyviral Protein 6K1 Reduces Plant Proteases Activity during Turnip mosaic virus Infection
Source: Viruses. 2022 Jun 20;14(6):1341. doi: 10.3390/v14061341 (PMC9229136; doi:10.3390/v14061341)
Supplement: Supplementary file 1 [file viruses-14-01341-s001.zip › viruses-1727107-supplementary.pdf]

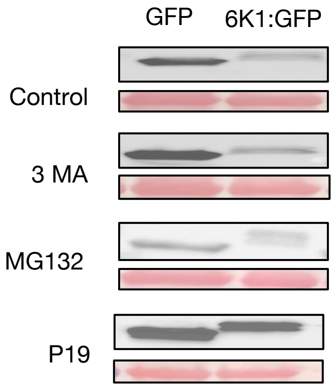

Figure S1. Western blot analysis of transiently expressed GFP and 6K1:GFP was assessed in the presence of chemical inhibitors of the autophagy protein degradation pathway
(3MA), the proteasome protein degradation pathway (MG132) and P19, an RNA interference silencing suppressor. Constructs were co-agroinfiltrated, while chemical inhibitors were infiltrated 48 hours after agroinfiltrations. For each sample, an equal volume was loaded into each well of an SDS-PAGE gel. Anti-GFP was used in both western blots and Ponceau staining was performed to check for loading control. All western blots are representative of at least two replicates which contained 3 plants per treatment.

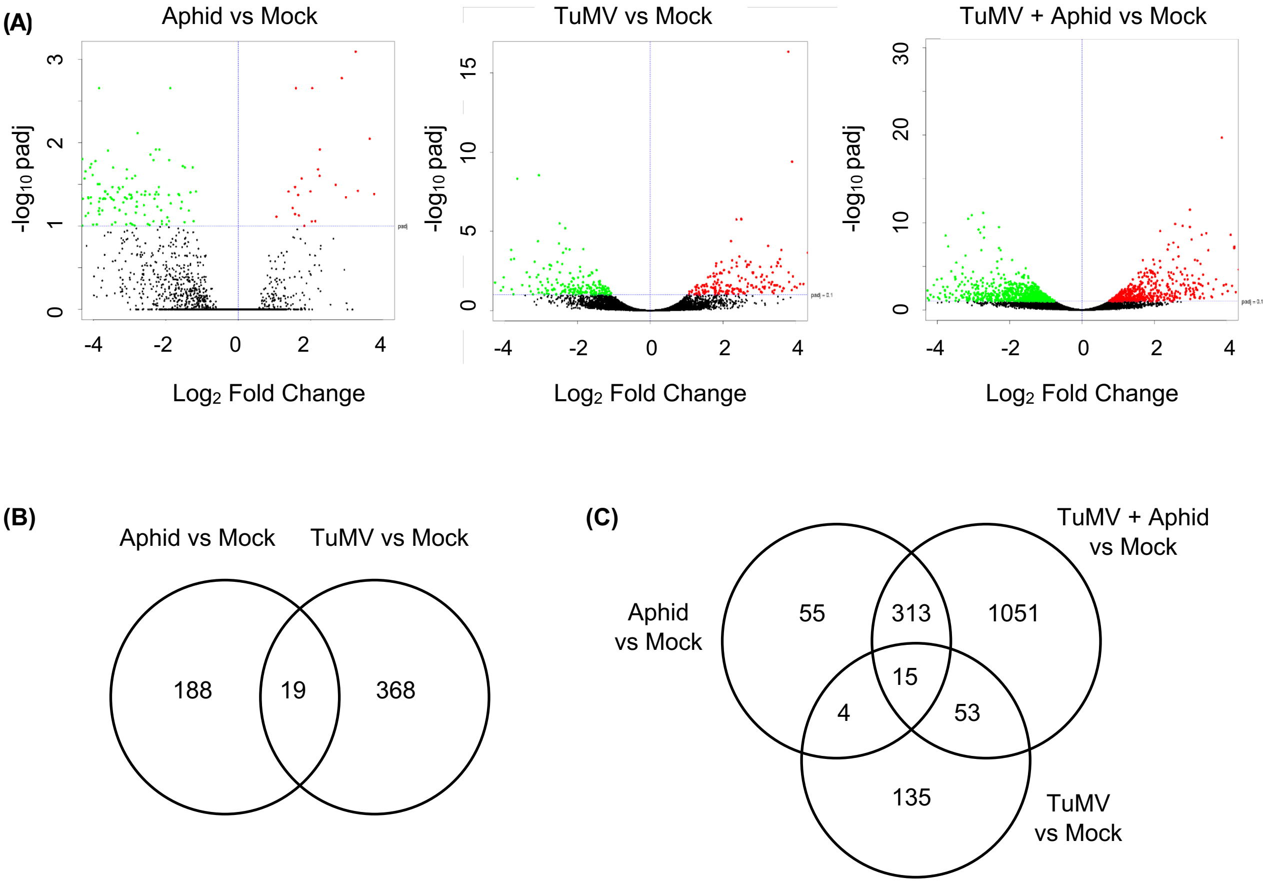

Fig. S2. (A) Volcano-plots of $-\log_{10}p$ and $\log_2FC$ exhibited by each gene in *Arabidopsis thaliana* with aphids, TuMV*, or* both aphids and TuMV, compared to mock controls. Up-and down-regulated genes are presented in red and green, respectively. (B) Numbers of differentially expressed genes (DEGs) shared between aphid-infested and TuMV-infected *A. thaliana* compared to mock controls. (C) Numbers of differentially expressed genes (DEGs) shared among all three treatments compared to mock controls. DEGs were identified using DESeq2and defined by $|\log2FC| \geq 1$; false discovery rate (FDR)-corrected p-value ≤ 0.1*.* FC, fold-change; p, FDR-corrected p-value.

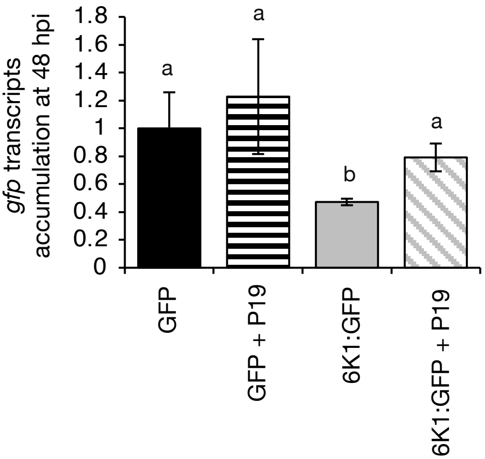

Fig. S3. Inhibition of the RNA silencing pathway by P19 increases the accumulation of *6K1:gfp* transcripts. GFP and 6K1:GFP were transiently expressed with/out P19 and *gfp* transcripts were quantified at 48 hr post infiltration. A general linear model was used to check for significant differences among means ± SE, letters indicate differences between means at a *P*-value of < 0.05.

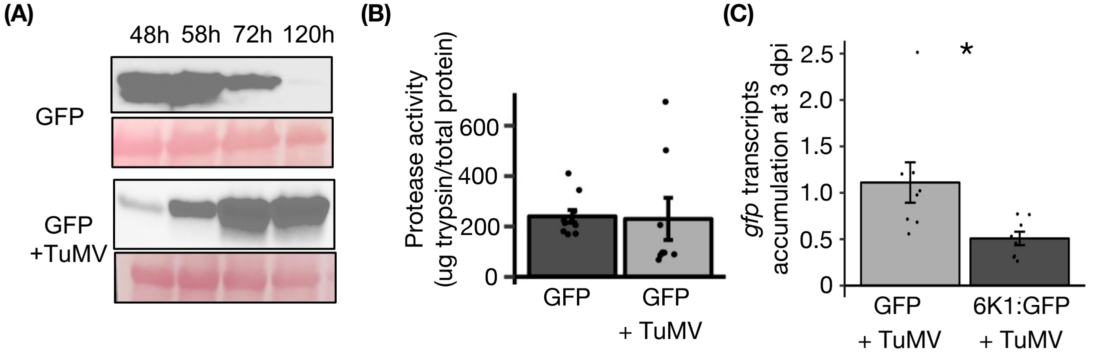

Fig. S4. TuMV increases the expression of GFP for longer time, but protease activity is not changed. (A) Western blots were performed with *Nicotiana benthamiana* leaves expressing GFP with and without TuMV over time and (B) protease activity was measured 120 hpi. For each sample, an equal volume (10µl) was loaded into each well of an SDS-PAGE gel. Anti-GFP was used in both western blots and Ponceau staining was performed as to check for loading control. (*n* = 10, mean ± SE; one-way ANOVA for B). (C) GFP and 6K1:GFP was transiently expressed with TuMV and *gfp* transcripts were quantified at 3 dpi post infiltration. (*n* = 8, mean ± SE; one-way ANOVA for C).

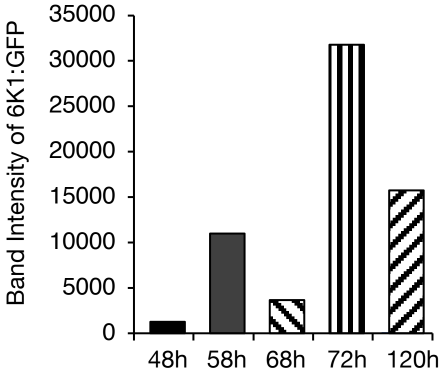

Fig. S5. A histogram indicating the protein band intensities observed in Fig. 4C .

Supplementary Table 1. A list of primers used for quantification.

Gene	Amplicon	Tm	Sequence (5' - 3')
Actin2F	108	58.2	GGTAACATTGTGCTCAGTGGTGG
Actin2R	108	55.7	AACGACCTTAATCTTCATGCTGC
NBR1F	152	58.4	CATTTTCTGGGGTGCCAAACGA
NBR1R	152	58.3	GAACCTAGGGCCAGTTATCGGA
cystatinF	157	57.9	CTC GCT TTG CTG TCG ATG AAC A
cystatinR	157	56.8	ACC TTG GCT TCG TAT GCT TTC T
Rpn11F	153	57.5	AAA GCA GTG CAG GAA GAG GAT G
Rpn11R	153	57.9	AAC TGT GTC AAG CAT GGT TCC C
lox(1)F	160	57.6	CTT TTG TGG GTT GTT GCT GCT G
lox(1)R	160	57.8	CTA AAA CTG CTC CAA CCT CCC C
lox(2)F	167	57.7	GGG GAT GCA GAG ATT GTC GAA G
lox(2)R	167	57.8	CAT GTT ACT CCA GGG CCA GAA C
TumvCpF	117	57.8	CAT TGA GAA CGG AAC CTC CCC
TumvCpR	117	58.6	TGC CTA AAT GTG GGT TTG GCG

Supplementary Table 2. Enriched Biological Processes (BPs) during the *A. thaliana* interaction with *Myzus persicae* aphids. Overrepresented BPs were identified based on a hypergeometric test with False Discovery Rate (FDR)-adjusted p-values ≤ 0.05. GO: Gene Ontology.

GO ID	GO term	P-value
GO:0009607	response to biotic stimulus	1.04E-13
GO:0009605	response to external stimulus	1.10E-12
GO:0009719	response to endogenous stimulus	2.89E-12
GO:0010033	response to organic substance	4.46E-12
GO:0009611	response to wounding	3.37E-11
GO:0009628	response to abiotic stimulus	4.67E-11
GO:0006952	defense response	3.26E-10
GO:0009753	response to jasmonic acid	2.75E-09
GO:0071456	cellular response to hypoxia	1.18E-08
GO:0071453	cellular response to oxygen levels	1.31E-08
GO:0036293	response to decreased oxygen levels	5.23E-08
GO:0009698	phenylpropanoid metabolic process	7.01E-08
GO:0044550	secondary metabolite biosynthetic process	1.64E-07
GO:0010035	response to inorganic substance	4.66E-07
GO:0009737	response to abscisic acid	3.54E-06
GO:0002376	immune system process	5.13E-06
GO:0042221	response to chemical	1.10E-05
GO:0009718	anthocyanin-containing compound biosynthetic process	1.15E-05
GO:0010345	suberin biosynthetic process	1.25E-05
GO:0009733	response to auxin	1.52E-05
GO:0051716	cellular response to stimulus	1.57E-05
GO:0042440	pigment metabolic process	1.68E-05
GO:0009414	response to water deprivation	1.97E-05
GO:0045087	innate immune response	2.88E-05
GO:0051707	response to other organism	4.81E-05
GO:0050832	defense response to fungus	6.40E-05
GO:1902609	(R)-2-hydroxy-alpha-linolenic acid biosynthetic process	6.76E-05
GO:0009751	response to salicylic acid	1.26E-04
GO:0009651	response to salt stress	1.43E-04
GO:0071495	cellular response to endogenous stimulus	3.20E-04
GO:0006950	response to stress	3.22E-04
GO:0007568	aging	3.74E-04
GO:0071395	cellular response to jasmonic acid stimulus	3.78E-04
GO:0031542	positive regulation of anthocyanin biosynthetic process	4.01E-04
GO:0009627	systemic acquired resistance	4.05E-04
GO:0042742	defense response to bacterium	5.96E-04
GO:0071446	cellular response to salicylic acid stimulus	6.47E-04
GO:0002213	defense response to insect	9.67E-04
GO:0010150	leaf senescence	9.73E-04
GO:0010230	alternative respiration	1.38E-03
GO:0071554	cell wall organization or biogenesis	1.52E-03
GO:0071310	cellular response to organic substance	1.54E-03
GO:0009867	jasmonic acid mediated signaling pathway	1.76E-03
GO:0035336	long-chain fatty-acyl-CoA metabolic process	1.83E-03
GO:0043449	cellular alkene metabolic process	1.90E-03
GO:1900674	olefin biosynthetic process	1.90E-03

GO:0009620	response to fungus	2.18E-03
GO:0009643	photosynthetic acclimation	2.34E-03
GO:0031540	regulation of anthocyanin biosynthetic process	2.85E-03
GO:0006869	lipid transport	2.98E-03
GO:0042545	cell wall modification	2.99E-03
GO:0010252	auxin homeostasis	3.22E-03
GO:0006979	response to oxidative stress	3.23E-03
GO:0072593	reactive oxygen species metabolic process	3.43E-03
GO:0080167	response to karrikin	4.21E-03
GO:0009813	flavonoid biosynthetic process	4.42E-03
GO:0006790	sulfur compound metabolic process	4.63E-03
GO:1901699	cellular response to nitrogen compound	4.67E-03
GO:0009624	response to nematode	4.95E-03
GO:0006637	acyl-CoA metabolic process	5.69E-03
GO:0001676	long-chain fatty acid metabolic process	5.76E-03
GO:0012501	programmed cell death	6.32E-03
GO:0044281	small molecule metabolic process	6.48E-03
GO:0006032	chitin catabolic process	6.61E-03
GO:0046348	amino sugar catabolic process	6.61E-03
GO:0080027	response to herbivore	6.61E-03
GO:0006949	syncytium formation	7.52E-03
GO:0016024	CDP-diacylglycerol biosynthetic process	7.52E-03
GO:0002239	response to oomycetes	7.52E-03
GO:0009664	plant-type cell wall organization	7.72E-03
GO:0032787	monocarboxylic acid metabolic process	7.74E-03
GO:0009809	lignin biosynthetic process	8.13E-03
GO:0009715	chalcone biosynthetic process	8.24E-03
GO:0090400	stress-induced premature senescence	8.24E-03
GO:1901038	cyanidin 3-O-glucoside metabolic process	8.24E-03
GO:1904250	positive regulation of age-related resistance	8.24E-03
GO:0000302	response to reactive oxygen species	9.08E-03
GO:0017144	drug metabolic process	9.26E-03
GO:0042445	hormone metabolic process	9.40E-03
GO:0042343	indole glucosinolate metabolic process	9.48E-03
GO:0009817	defense response to fungus, incompatible interaction	9.54E-03
GO:0065008	regulation of biological quality	1.13E-02
GO:0045088	regulation of innate immune response	1.16E-02
GO:0050778	positive regulation of immune response	1.16E-02
GO:0006022	aminoglycan metabolic process	1.16E-02
GO:0010393	galacturonan metabolic process	1.17E-02
GO:0009723	response to ethylene	1.20E-02
GO:0009863	salicylic acid mediated signaling pathway	1.34E-02
GO:0033865	nucleoside bisphosphate metabolic process	1.34E-02
GO:1901700	response to oxygen-containing compound	1.46E-02
GO:0016042	lipid catabolic process	1.52E-02
GO:0010143	cutin biosynthetic process	1.53E-02
GO:1901071	glucosamine-containing compound metabolic process	1.53E-02
GO:0045229	external encapsulating structure organization	1.61E-02
GO:0001561	fatty acid alpha-oxidation	1.64E-02
GO:0010055	atrichoblast differentiation	1.64E-02
GO:0010365	positive regulation of ethylene biosynthetic process	1.64E-02
GO:0015760	glucose-6-phosphate transport	1.64E-02

GO:1900030	regulation of pectin biosynthetic process	1.64E-02
GO:1900910	positive regulation of olefin metabolic process	1.64E-02
GO:1901064	syringal lignin metabolic process	1.64E-02
GO:1901430	positive regulation of syringal lignin biosynthetic process	1.64E-02
GO:2000029	regulation of proanthocyanidin biosynthetic process	1.64E-02
GO:0016311	dephosphorylation	1.65E-02
GO:0018958	phenol-containing compound metabolic process	1.66E-02
GO:0008300	isoprenoid catabolic process	1.79E-02
GO:0010167	response to nitrate	1.79E-02
GO:0019438	aromatic compound biosynthetic process	1.85E-02
GO:0044262	cellular carbohydrate metabolic process	1.92E-02
GO:0033559	unsaturated fatty acid metabolic process	1.93E-02
GO:0002682	regulation of immune system process	1.95E-02
GO:0019757	glycosinolate metabolic process	2.00E-02
GO:1901615	organic hydroxy compound metabolic process	2.12E-02
GO:0009693	ethylene biosynthetic process	2.20E-02
GO:0010224	response to UV-B	2.25E-02
GO:0009409	response to cold	2.29E-02
GO:0009744	response to sucrose	2.33E-02
GO:0009636	response to toxic substance	2.34E-02
GO:0002833	positive regulation of response to biotic stimulus	2.38E-02
GO:0009745	sucrose mediated signaling	2.45E-02
GO:0009830	cell wall modification involved in abscission	2.45E-02
GO:0010597	green leaf volatile biosynthetic process	2.45E-02
GO:0015824	proline transport	2.45E-02
GO:0016110	tetraterpenoid catabolic process	2.45E-02
GO:0016124	xanthophyll catabolic process	2.45E-02
GO:0019477	L-lysine catabolic process	2.45E-02
GO:0019878	lysine biosynthetic process via aminoadipic acid	2.45E-02
GO:0032491	detection of molecule of fungal origin	2.45E-02
GO:0033512	L-lysine catabolic process to acetyl-CoA via saccharopine	2.45E-02
GO:0045764	positive regulation of cellular amino acid metabolic process	2.45E-02
GO:0048838	release of seed from dormancy	2.45E-02
GO:0060774	auxin mediated signaling pathway involved in phyllotactic patterning	2.45E-02
GO:0071497	cellular response to freezing	2.45E-02
GO:0043436	oxoacid metabolic process	2.51E-02
GO:0009682	induced systemic resistance	2.53E-02
GO:0010200	response to chitin	2.58E-02
GO:0032103	positive regulation of response to external stimulus	2.63E-02
GO:1901362	organic cyclic compound biosynthetic process	2.77E-02
GO:0001101	response to acid chemical	3.08E-02
GO:0009117	nucleotide metabolic process	3.11E-02
GO:0010383	cell wall polysaccharide metabolic process	3.15E-02
GO:0031349	positive regulation of defense response	3.19E-02
GO:0010029	regulation of seed germination	3.23E-02
GO:0080134	regulation of response to stress	3.25E-02
GO:0002240	response to molecule of oomycetes origin	3.26E-02
GO:0006690	icosanoid metabolic process	3.26E-02
GO:0015692	lead ion transport	3.26E-02
GO:0015714	phosphoenolpyruvate transport	3.26E-02
GO:0016137	glycoside metabolic process	3.26E-02
GO:0035264	multicellular organism growth	3.26E-02

GO:0035436	triose phosphate transmembrane transport	3.26E-02
GO:0042218	1-aminocyclopropane-1-carboxylate biosynthetic process	3.26E-02
GO:0060772	leaf phyllotactic patterning	3.26E-02
GO:0071732	cellular response to nitric oxide	3.26E-02
GO:0002237	response to molecule of bacterial origin	3.54E-02
GO:0006817	phosphate ion transport	3.54E-02
GO:0008219	cell death	3.58E-02
GO:0048827	phyllome development	3.72E-02
GO:0009828	plant-type cell wall loosening	3.73E-02
GO:1905039	carboxylic acid transmembrane transport	3.74E-02
GO:0007569	cell aging	4.05E-02
GO:0009413	response to flooding	4.05E-02
GO:0019516	lactate oxidation	4.05E-02
GO:0032107	regulation of response to nutrient levels	4.05E-02
GO:0051091	positive regulation of DNA-binding transcription factor activity	4.05E-02
GO:0051410	detoxification of nitrogen compound	4.05E-02
GO:0071323	cellular response to chitin	4.05E-02
GO:0097366	response to bronchodilator	4.05E-02
GO:1900384	regulation of flavonol biosynthetic process	4.05E-02
GO:1901334	lactone metabolic process	4.05E-02
GO:1901601	strigolactone biosynthetic process	4.05E-02
GO:0006820	anion transport	4.17E-02
GO:0071365	cellular response to auxin stimulus	4.28E-02
GO:0009743	response to carbohydrate	4.28E-02
GO:0046395	carboxylic acid catabolic process	4.45E-02
GO:2000022	regulation of jasmonic acid mediated signaling pathway	4.49E-02
GO:0008643	carbohydrate transport	4.51E-02
GO:0045490	pectin catabolic process	4.51E-02
GO:0044282	small molecule catabolic process	4.76E-02
GO:0010023	proanthocyanidin biosynthetic process	4.82E-02
GO:0015713	phosphoglycerate transmembrane transport	4.85E-02
GO:0043903	regulation of interspecies interactions between organisms	4.85E-02
GO:0046345	abscisic acid catabolic process	4.85E-02
GO:0080144	amino acid homeostasis	4.85E-02
GO:0080187	floral organ senescence	4.85E-02
GO:1900367	positive regulation of defense response to insect	4.85E-02
GO:1901672	positive regulation of systemic acquired resistance	4.85E-02
GO:0010043	response to zinc ion	4.90E-02

Supplementary Table 3. Enriched BPs during the *A. thaliana* interaction with TuMV. Overrepresented BPs were identified based on a hypergeometric test with False Discovery Rate (FDR)-adjusted p-values ≤ 0.05. GO: Gene Ontology.

GO ID	GO term	P-value
GO:0006952	defense response	2.41E-07
GO:0009607	response to biotic stimulus	2.57E-07
GO:0051707	response to other organism	3.59E-07
GO:0071456	cellular response to hypoxia	9.14E-06
GO:0071453	cellular response to oxygen levels	1.00E-05
GO:0009605	response to external stimulus	1.30E-05
GO:0050896	response to stimulus	1.42E-05
GO:0036293	response to decreased oxygen levels	3.44E-05
GO:0009628	response to abiotic stimulus	1.04E-04
GO:0050832	defense response to fungus	1.27E-04
GO:0010150	leaf senescence	1.60E-04
GO:0009627	systemic acquired resistance	1.73E-04
GO:0006979	response to oxidative stress	8.46E-04
GO:0010200	response to chitin	9.74E-04
GO:0002376	immune system process	1.05E-03
GO:1901700	response to oxygen-containing compound	1.24E-03
GO:0006949	syncytium formation	1.41E-03
GO:0046395	carboxylic acid catabolic process	2.26E-03
GO:0045087	innate immune response	2.42E-03
GO:0007568	aging	2.58E-03
GO:0046345	abscisic acid catabolic process	2.94E-03
GO:0048830	adventitious root development	2.94E-03
GO:1901698	response to nitrogen compound	3.53E-03
GO:0010230	alternative respiration	4.08E-03
GO:0043090	amino acid import	4.08E-03
GO:0002229	defense response to oomycetes	5.20E-03
GO:0010258	NADH dehydrogenase complex (plastoquinone) assembly	5.38E-03
GO:0048629	trichome patterning	5.38E-03
GO:0009664	plant-type cell wall organization	5.48E-03
GO:0042221	response to chemical	6.29E-03
GO:0006730	one-carbon metabolic process	6.56E-03
GO:0001101	response to acid chemical	6.67E-03
GO:0044282	small molecule catabolic process	7.47E-03
GO:0009611	response to wounding	8.01E-03
GO:0009610	response to symbiotic fungus	8.49E-03
GO:0002213	defense response to insect	8.82E-03
GO:0006563	L-serine metabolic process	8.82E-03
GO:0009631	cold acclimation	8.92E-03
GO:0042538	hyperosmotic salinity response	9.47E-03
GO:0010411	xyloglucan metabolic process	1.00E-02
GO:0006538	glutamate catabolic process	1.03E-02
GO:0006586	indolalkylamine metabolic process	1.15E-02
GO:0044042	glucan metabolic process	1.18E-02
GO:0009819	drought recovery	1.22E-02
GO:0048827	phyllome development	1.22E-02
GO:0070417	cellular response to cold	1.25E-02

GO:0010583	response to cyclopentenone	1.35E-02
GO:0016145	S-glycoside catabolic process	1.35E-02
GO:0019762	glucosinolate catabolic process	1.35E-02
GO:0010033	response to organic substance	1.36E-02
GO:0000495	box H/ACA snoRNA 3'-end processing	1.43E-02
GO:0009667	plastid inner membrane organization	1.43E-02
GO:0009835	fruit ripening	1.43E-02
GO:0033979	box H/ACA snoRNA metabolic process	1.43E-02
GO:0042866	pyruvate biosynthetic process	1.43E-02
GO:0045176	apical protein localization	1.43E-02
GO:0048498	establishment of petal orientation	1.43E-02
GO:0080149	sucrose induced translational repression	1.43E-02
GO:0090042	tubulin deacetylation	1.43E-02
GO:0090428	perianth development	1.43E-02
GO:0090707	establishment of plant organ orientation	1.43E-02
GO:1902455	negative regulation of stem cell population maintenance	1.43E-02
GO:0042436	indole-containing compound catabolic process	1.43E-02
GO:0061077	chaperone-mediated protein folding	1.47E-02
GO:0009828	plant-type cell wall loosening	1.57E-02
GO:0002238	response to molecule of fungal origin	1.64E-02
GO:0009617	response to bacterium	1.74E-02
GO:0042493	response to drug	1.82E-02
GO:0051716	cellular response to stimulus	1.90E-02
GO:0009409	response to cold	1.93E-02
GO:0009861	jasmonic acid and ethylene-dependent systemic resistance	2.14E-02
GO:0016115	terpenoid catabolic process	2.14E-02
GO:0044003	modulation by symbiont of host process	2.40E-02
GO:0009753	response to jasmonic acid	2.47E-02
GO:1901616	organic hydroxy compound catabolic process	2.49E-02
GO:0031347	regulation of defense response	2.61E-02
GO:0006970	response to osmotic stress	2.64E-02
GO:0010555	response to mannitol	2.68E-02
GO:0035821	modulation of process of other organism	2.68E-02
GO:0009725	response to hormone	2.82E-02
GO:0002164	larval development	2.84E-02
GO:0008153	para-aminobenzoic acid biosynthetic process	2.84E-02
GO:0009609	response to symbiotic bacterium	2.84E-02
GO:0043555	regulation of translation in response to stress	2.84E-02
GO:0046244	salicylic acid catabolic process	2.84E-02
GO:0061062	regulation of nematode larval development	2.84E-02
GO:0070179	D-serine biosynthetic process	2.84E-02
GO:0072702	response to methyl methanesulfonate	2.84E-02
GO:1900030	regulation of pectin biosynthetic process	2.84E-02
GO:1990966	ATP generation from poly-ADP-D-ribose	2.84E-02
GO:2000905	negative regulation of starch metabolic process	2.84E-02
GO:0043144	snoRNA processing	2.97E-02
GO:0010035	response to inorganic substance	2.98E-02
GO:0044106	cellular amine metabolic process	3.00E-02
GO:1901136	carbohydrate derivative catabolic process	3.12E-02
GO:0009612	response to mechanical stimulus	3.26E-02
GO:0044403	symbiotic process	3.28E-02
GO:0009072	aromatic amino acid family metabolic process	3.36E-02

GO:0002831	regulation of response to biotic stimulus	3.40E-02
GO:0003008	system process	3.57E-02
GO:0003018	vascular process in circulatory system	3.57E-02
GO:0010233	phloem transport	3.57E-02
GO:1901606	alpha-amino acid catabolic process	3.61E-02
GO:0032101	regulation of response to external stimulus	3.84E-02
GO:0044036	cell wall macromolecule metabolic process	3.84E-02
GO:0045229	external encapsulating structure organization	3.86E-02
GO:0140115	export across plasma membrane	3.90E-02
GO:0043436	oxoacid metabolic process	3.97E-02
GO:0051607	defense response to virus	4.01E-02
GO:0015979	photosynthesis	4.09E-02
GO:0009414	response to water deprivation	4.14E-02
GO:0000256	allantoin catabolic process	4.23E-02
GO:0000494	box C/D snoRNA 3'-end processing	4.23E-02
GO:0009915	phloem sucrose loading	4.23E-02
GO:0015824	proline transport	4.23E-02
GO:0031120	snRNA pseudouridine synthesis	4.23E-02
GO:0032491	detection of molecule of fungal origin	4.23E-02
GO:0033194	response to hydroperoxide	4.23E-02
GO:0033967	box C/D snoRNA metabolic process	4.23E-02
GO:0048838	release of seed from dormancy	4.23E-02
GO:0051973	positive regulation of telomerase activity	4.23E-02
GO:0062034	L-pipecolic acid biosynthetic process	4.23E-02
GO:0072718	response to cisplatin	4.23E-02
GO:0090357	regulation of tryptophan metabolic process	4.23E-02
GO:1990258	histone glutamine methylation	4.23E-02
GO:0071365	cellular response to auxin stimulus	4.59E-02
GO:0009064	glutamine family amino acid metabolic process	4.80E-02
GO:0007165	signal transduction	4.83E-02
GO:0009416	response to light stimulus	4.90E-02
GO:0042026	protein refolding	4.92E-02

Supplementary Table 4. Enriched Biological Processes (BPs) during the *A. thaliana* interaction with a combination of both *M. persicae* aphids and TuMV. Overrepresented BPs were identified based on a hypergeometric test with False Discovery Rate (FDR)-adjusted p-values ≤ 0.05. GO: Gene Ontology.

GO ID	GO term	P-value
GO:0044419	interspecies interaction between organisms	7.57E-22
GO:0043207	response to external biotic stimulus	4.13E-20
GO:0009628	response to abiotic stimulus	5.24E-17
GO:0010033	response to organic substance	5.79E-12
GO:0009611	response to wounding	8.11E-10
GO:0009719	response to endogenous stimulus	3.04E-09
GO:0019748	secondary metabolic process	9.66E-09
GO:0009751	response to salicylic acid	1.03E-07
GO:0042742	defense response to bacterium	1.27E-07
GO:0009753	response to jasmonic acid	2.05E-07
GO:0050832	defense response to fungus	2.10E-07
GO:0009414	response to water deprivation	2.15E-07
GO:0010035	response to inorganic substance	2.35E-07
GO:0006955	immune response	4.16E-07
GO:0036293	response to decreased oxygen levels	7.27E-07
GO:0071456	cellular response to hypoxia	1.13E-06
GO:0071453	cellular response to oxygen levels	1.36E-06
GO:0009718	anthocyanin-containing compound biosynthetic process	1.57E-06
GO:0009409	response to cold	1.87E-06
GO:0019684	photosynthesis, light reaction	2.44E-06
GO:0080167	response to karrikin	3.42E-06
GO:0051716	cellular response to stimulus	5.44E-06
GO:0009631	cold acclimation	6.41E-06
GO:1901700	response to oxygen-containing compound	7.18E-06
GO:0009664	plant-type cell wall organization	1.05E-05
GO:0010200	response to chitin	1.61E-05
GO:0042537	benzene-containing compound metabolic process	1.71E-05
GO:0060866	leaf abscission	1.99E-05
GO:0016053	organic acid biosynthetic process	3.41E-05
GO:1901617	organic hydroxy compound biosynthetic process	4.02E-05
GO:0042538	hyperosmotic salinity response	4.11E-05
GO:0050821	protein stabilization	6.76E-05
GO:0009627	systemic acquired resistance	9.04E-05
GO:0044281	small molecule metabolic process	9.94E-05
GO:0046345	abscisic acid catabolic process	1.06E-04
GO:0009773	photosynthetic electron transport in photosystem I	1.08E-04
GO:0044255	cellular lipid metabolic process	1.09E-04
GO:0018958	phenol-containing compound metabolic process	1.32E-04
GO:0001101	response to acid chemical	1.77E-04
GO:0042221	response to chemical	1.99E-04
GO:1901698	response to nitrogen compound	2.00E-04
GO:0009636	response to toxic substance	2.82E-04
GO:0006979	response to oxidative stress	2.89E-04
GO:0042440	pigment metabolic process	4.39E-04
GO:0010258	NADH dehydrogenase complex (plastoquinone) assembly	4.55E-04
GO:0006950	response to stress	6.15E-04

GO:0044038	cell wall macromolecule biosynthetic process	6.18E-04
GO:0043436	oxoacid metabolic process	6.71E-04
GO:0033692	cellular polysaccharide biosynthetic process	6.84E-04
GO:0010817	regulation of hormone levels	7.69E-04
GO:0044262	cellular carbohydrate metabolic process	8.24E-04
GO:0010150	leaf senescence	8.98E-04
GO:0005975	carbohydrate metabolic process	9.77E-04
GO:0080134	regulation of response to stress	1.03E-03
GO:0006949	syncytium formation	1.09E-03
GO:0016115	terpenoid catabolic process	1.09E-03
GO:0002213	defense response to insect	1.25E-03
GO:0016999	antibiotic metabolic process	1.38E-03
GO:0098542	defense response to other organism	1.40E-03
GO:0043288	apocarotenoid metabolic process	1.43E-03
GO:1902644	tertiary alcohol metabolic process	1.43E-03
GO:0002831	regulation of response to biotic stimulus	1.43E-03
GO:0002238	response to molecule of fungal origin	1.47E-03
GO:0009737	response to abscisic acid	1.59E-03
GO:0050896	response to stimulus	1.79E-03
GO:0015979	photosynthesis	1.85E-03
GO:0010190	cytochrome b6f complex assembly	1.89E-03
GO:0080142	regulation of salicylic acid biosynthetic process	1.89E-03
GO:0019752	carboxylic acid metabolic process	1.92E-03
GO:0032101	regulation of response to external stimulus	2.01E-03
GO:0046395	carboxylic acid catabolic process	2.14E-03
GO:0006714	sesquiterpenoid metabolic process	2.21E-03
GO:0009416	response to light stimulus	2.29E-03
GO:0016125	sterol metabolic process	2.29E-03
GO:0071229	cellular response to acid chemical	2.41E-03
GO:0045488	pectin metabolic process	2.46E-03
GO:0010306	rhamnogalacturonan II biosynthetic process	2.60E-03
GO:0048830	adventitious root development	2.60E-03
GO:0090391	granum assembly	2.60E-03
GO:0006633	fatty acid biosynthetic process	2.62E-03
GO:0006629	lipid metabolic process	2.62E-03
GO:0006749	glutathione metabolic process	2.81E-03
GO:0009828	plant-type cell wall loosening	2.86E-03
GO:0010383	cell wall polysaccharide metabolic process	3.12E-03
GO:0002239	response to oomycetes	3.16E-03
GO:0050829	defense response to Gram-negative bacterium	3.23E-03
GO:0009867	jasmonic acid mediated signaling pathway	3.24E-03
GO:0009407	toxin catabolic process	3.84E-03
GO:0009699	phenylpropanoid biosynthetic process	4.21E-03
GO:0052386	cell wall thickening	4.24E-03
GO:0009697	salicylic acid biosynthetic process	4.33E-03
GO:0044042	glucan metabolic process	4.45E-03
GO:0030091	protein repair	5.01E-03
GO:0048827	phyllome development	5.72E-03
GO:0002682	regulation of immune system process	5.82E-03
GO:0009625	response to insect	5.92E-03
GO:0009768	photosynthesis, light harvesting in photosystem I	6.15E-03
GO:0010112	regulation of systemic acquired resistance	6.15E-03

GO:0010540	basipetal auxin transport	6.59E-03
GO:0080027	response to herbivore	6.59E-03
GO:0009759	indole glucosinolate biosynthetic process	6.72E-03
GO:0009617	response to bacterium	6.82E-03
GO:0007568	aging	6.83E-03
GO:0071446	cellular response to salicylic acid stimulus	6.85E-03
GO:0042430	indole-containing compound metabolic process	7.09E-03
GO:1901616	organic hydroxy compound catabolic process	7.73E-03
GO:0044282	small molecule catabolic process	7.76E-03
GO:0048825	cotyledon development	7.85E-03
GO:1901701	cellular response to oxygen-containing compound	8.03E-03
GO:0010412	mannan metabolic process	8.05E-03
GO:0010618	aerenchyma formation	8.05E-03
GO:0019695	choline metabolic process	8.05E-03
GO:0033614	chloroplast proton-transporting ATP synthase complex assembly	8.05E-03
GO:0045943	positive regulation of transcription by RNA polymerase I	8.05E-03
GO:0048838	release of seed from dormancy	8.05E-03
GO:0052544	defense response by callose deposition in cell wall	8.42E-03
GO:0016051	carbohydrate biosynthetic process	8.54E-03
GO:0006694	steroid biosynthetic process	8.75E-03
GO:0042026	protein refolding	8.90E-03
GO:0009408	response to heat	9.85E-03
GO:0055114	oxidation-reduction process	1.04E-02
GO:0031349	positive regulation of defense response	1.05E-02
GO:0104004	cellular response to environmental stimulus	1.13E-02
GO:0072330	monocarboxylic acid biosynthetic process	1.17E-02
GO:0009644	response to high light intensity	1.18E-02
GO:0010411	xyloglucan metabolic process	1.19E-02
GO:0019252	starch biosynthetic process	1.22E-02
GO:0007165	signal transduction	1.25E-02
GO:0071555	cell wall organization	1.27E-02
GO:0009620	response to fungus	1.28E-02
GO:0010588	cotyledon vascular tissue pattern formation	1.30E-02
GO:0031407	oxylipin metabolic process	1.30E-02
GO:0052325	cell wall pectin biosynthetic process	1.32E-02
GO:0010942	positive regulation of cell death	1.45E-02
GO:0010136	ureide catabolic process	1.55E-02
GO:0048281	inflorescence morphogenesis	1.55E-02
GO:1901562	response to paraquat	1.55E-02
GO:1902479	positive regulation of defense response to bacterium, incompatible interaction	1.55E-02
GO:0009651	response to salt stress	1.62E-02
GO:0000302	response to reactive oxygen species	1.63E-02
GO:0005976	polysaccharide metabolic process	1.65E-02
GO:0016132	brassinosteroid biosynthetic process	1.67E-02
GO:0010431	seed maturation	1.69E-02
GO:0009808	lignin metabolic process	1.73E-02
GO:0006952	defense response	1.83E-02
GO:0019758	glycosinolate biosynthetic process	1.88E-02
GO:1900426	positive regulation of defense response to bacterium	1.89E-02
GO:0009612	response to mechanical stimulus	1.90E-02
GO:0009682	induced systemic resistance	1.92E-02
GO:0001505	regulation of neurotransmitter levels	1.97E-02

GO:0009813	flavonoid biosynthetic process	2.12E-02
GO:0009934	regulation of meristem structural organization	2.25E-02
GO:0015740	C4-dicarboxylate transport	2.25E-02
GO:0060429	epithelium development	2.25E-02
GO:0009684	indoleacetic acid biosynthetic process	2.25E-02
GO:0006468	protein phosphorylation	2.41E-02
GO:0010565	regulation of cellular ketone metabolic process	2.47E-02
GO:0006145	purine nucleobase catabolic process	2.50E-02
GO:0006883	cellular sodium ion homeostasis	2.50E-02
GO:0009413	response to flooding	2.50E-02
GO:0019218	regulation of steroid metabolic process	2.50E-02
GO:0034087	establishment of mitotic sister chromatid cohesion	2.50E-02
GO:0045922	negative regulation of fatty acid metabolic process	2.50E-02
GO:0070550	rDNA condensation	2.50E-02
GO:0071169	establishment of protein localization to chromatin	2.50E-02
GO:0071733	transcriptional activation by promoter-enhancer looping	2.50E-02
GO:0090579	dsDNA loop formation	2.50E-02
GO:0120187	positive regulation of protein localization to chromatin	2.50E-02
GO:1900378	positive regulation of secondary metabolite biosynthetic process	2.50E-02
GO:1905406	positive regulation of mitotic cohesin loading	2.50E-02
GO:1990414	replication-born double-strand break repair via sister chromatid exchange	2.50E-02
GO:0015766	disaccharide transport	2.64E-02
GO:0032870	cellular response to hormone stimulus	2.75E-02
GO:0006568	tryptophan metabolic process	2.81E-02
GO:0050778	positive regulation of immune response	2.96E-02
GO:0009624	response to nematode	3.06E-02
GO:0016108	tetraterpenoid metabolic process	3.16E-02
GO:0016128	phytosteroid metabolic process	3.16E-02
GO:0070417	cellular response to cold	3.16E-02
GO:0042446	hormone biosynthetic process	3.17E-02
GO:1901606	alpha-amino acid catabolic process	3.27E-02
GO:0002229	defense response to oomycetes	3.27E-02
GO:0044272	sulfur compound biosynthetic process	3.34E-02
GO:0031540	regulation of anthocyanin biosynthetic process	3.45E-02
GO:0045840	positive regulation of mitotic nuclear division	3.45E-02
GO:0010268	brassinosteroid homeostasis	3.52E-02
GO:0010583	response to cyclopentenone	3.53E-02
GO:0016145	S-glycoside catabolic process	3.53E-02
GO:0019762	glucosinolate catabolic process	3.53E-02
GO:0042181	ketone biosynthetic process	3.53E-02
GO:0042631	cellular response to water deprivation	3.53E-02
GO:0009957	epidermal cell fate specification	3.62E-02
GO:0010021	amylopectin biosynthetic process	3.62E-02
GO:0071486	cellular response to high light intensity	3.62E-02
GO:1900367	positive regulation of defense response to insect	3.62E-02
GO:0009312	oligosaccharide biosynthetic process	3.93E-02
GO:0048598	embryonic morphogenesis	3.93E-02
GO:1901568	fatty acid derivative metabolic process	3.95E-02
GO:0009615	response to virus	3.97E-02
GO:0006636	unsaturated fatty acid biosynthetic process	4.03E-02
GO:0034605	cellular response to heat	4.09E-02
GO:0005992	trehalose biosynthetic process	4.14E-02

GO:0009862	systemic acquired resistance, salicylic acid mediated signaling pathway	4.14E-02
GO:0010206	photosystem II repair	4.14E-02
GO:0010310	regulation of hydrogen peroxide metabolic process	4.14E-02
GO:0016104	triterpenoid biosynthetic process	4.14E-02
GO:0051085	chaperone cofactor-dependent protein refolding	4.31E-02
GO:0071470	cellular response to osmotic stress	4.31E-02
GO:0010118	stomatal movement	4.32E-02
GO:0009626	plant-type hypersensitive response	4.41E-02
GO:0010087	phloem or xylem histogenesis	4.53E-02
GO:0016117	carotenoid biosynthetic process	4.57E-02
GO:0009561	megagametogenesis	4.66E-02
GO:0016143	S-glycoside metabolic process	4.70E-02
GO:0019760	glucosinolate metabolic process	4.70E-02
GO:0009657	plastid organization	4.86E-02
GO:0009814	defense response, incompatible interaction	4.88E-02
GO:0006021	inositol biosynthetic process	4.89E-02
GO:0016120	carotene biosynthetic process	4.89E-02
GO:0019464	glycine decarboxylation via glycine cleavage system	4.89E-02
GO:0032210	regulation of telomere maintenance via telomerase	4.89E-02
GO:0042759	long-chain fatty acid biosynthetic process	4.89E-02
GO:0043090	amino acid import	4.89E-02
GO:0045876	positive regulation of sister chromatid cohesion	4.89E-02
GO:0060964	regulation of gene silencing by miRNA	4.89E-02
GO:0071922	regulation of cohesin loading	4.89E-02
GO:1902290	positive regulation of defense response to oomycetes	4.89E-02
GO:0009071	serine family amino acid catabolic process	4.90E-02
GO:0030497	fatty acid elongation	4.90E-02
